# When should coccidioidomycosis be in the differential?

**DOI:** 10.1128/asmcr.00052-25

**Published:** 2025-05-07

**Authors:** John N. Galgiani

**Affiliations:** 1 Valley Fever Center for Excellence, University of Arizona, College of Medicine-Tucson 733661, Tucson, Arizona, USA; 2 Department of Medicine, College of Medicine-Tucson, University of Arizona 242723, Tucson, Arizona, USA; 3 Department of Immunobiology, College of Medicine-Tucson, University of Arizona 242724, Tucson, Arizona, USA; 4 BIO5 Institute, University of Arizona 124486, Tucson, Arizona, USA; Vanderbilt University Medical Center, Nashville, Tennessee, USA

## Abstract

In a recent article in *ASM Case Reports*, Blomquist et al. (1:e00098-24, https://doi.org/10.1128/asmcr.00098-24) describe a young woman with several years of abdominal symptoms who subsequently was found to have peritoneal coccidioidomycosis. Her story calls into focus the value of considering this endemic disease, either in patients with typical or unusual manifestations. In this patient, eventually the concern for metastatic cancer led to a surgical procedure which established the true diagnosis. However, a simple serologic test, either done in the preceding years because of her chronic symptoms or shortly before her biopsy, would have improved her management. More generally, persons living in coccidioidal endemic regions who are diagnosed with community-acquired pneumonia, a common presentation of coccidioidomycosis, are often managed without being tested for this possibility. In addition, as with the patient described in the case report, many patients present with a variety of other syndromes, making it reasonable to include coccidioidomycosis in the differential diagnosis of a wider spectrum of illnesses where the exact diagnosis is not yet firmly established. These considerations are equally applicable to patients seen elsewhere with recent history of travel to *Coccidioides*-endemic locations.

## COMMENTARY

The case report by Blomquist et al. (1) describes a woman found to have coccidioidomycosis disseminated throughout her peritoneum, diagnosed by histopathology obtained during a peritoneoscopy once a computed tomography (CT) scan of the abdomen demonstrated ascites. The patient had had 6 years of abdominal complaints, and in the preceding month, the symptoms of night sweats, cough, dyspnea, and a 10 pound weight loss. In addition to abdominal findings, the CT scan also showed a right pulmonary nodule and a pleural effusion. Although not stated, the pleural effusion may have been on the same side as the pulmonary nodule since peripneumonic effusions are relatively common following initial coccidioidal infections (2). The authors emphasize that the exact duration of this infection could have been several weeks based upon the more recent symptoms or several years based upon the chronicity of the abdominal complaints. In either case, throughout the evaluation that led to the peritoneoscopy, coccidioidomycosis was not considered as a possible etiology. Coccidioidal antibodies are usually detectable in patients with disseminated infection, and this patient’s serum complement fixing antibody titer was strikingly high (1:256), obtained only after the biopsy results were known.

Peritoneal coccidioidomycosis is uncommon but not rare where coccidioidomycosis is endemic. This case report originated from Arizona, where I practice. From my own experience and in discussions with my colleagues, I would estimate that this complication happens to approximately 10–20 Arizona patients per year. Certainly, malignancy is a much more common cause of peritoneal nodules in women. For example, the American Cancer Society estimates that in 2024, the number of Arizona ovarian cancers was 6,830 (https://www.cancer.org/content/dam/cancer-org/research/cancer-facts-and-statistics/annual-cancer-facts-and-figures/2024/sd3-21-cancers-by-state-2024.pdf), a decidedly larger number. However, less than 5% of ovarian cancers occur in this patient’s age group (same web link), which reduces the ratio considerably. Given these statistics and even if the coccidioidal serology was known to be positive, obtaining tissue confirmation is likely needed to definitely exclude malignancy once the CT findings were known. However, knowing that coccidioidomycosis was a possible etiology might have been valuable information for the patient, providing her with hope for an alternative diagnosis to cancer, ahead of the procedure.

In Arizona and California, where most cases of coccidioidomycosis originate (3), there were over 25,000 reports to the Centers for Disease Control and Prevention (CDC) of this infection in 2024. This is the highest annual number on record. While this recent peak is likely part of year-to-year variations (4), long-term forecasts suggest that the endemic region for coccidioidomycosis will cover much of the Western United States by the end of the century (5). Serologic tests are the basis for the overwhelming proportion of new diagnoses, although cultures and histopathology from biopsies, as employed in this patient initially, and other approaches are also useful occasionally (6, 7). As the authors indicate, these statistics greatly underestimate the true numbers of patients who become ill. Obviously, it is difficult to know precisely how great the underestimate is. Recent attempts by the CDC to determine the true number of illnesses conclude that it is 10- to 18-fold greater than what is reported nationally (S. L. Williams, B. R. Jackson, K. Benedict, M. Rajeev, G. Cooksey, I. Ruberto, T. Williamson, R. Kuran, N. C. Bahr, L. Medina-Garcia, R. R. Reik, I. S. Schwartz, A. Carey, A. Spec, M. S. Freedman, R. H. Sunenshine, H. N. Oltean, and B. Osborn, unpublished data). Some infections go undiagnosed in persons after visiting an endemic region and who become ill only after returning home where coccidioidomycosis is uncommon. If that travel history is not obtained, testing for coccidioidomycosis would not be appropriate, so checking for travel exposure would be essential. Other cases not included in the statistics are those diagnosed in the 22 states and the District of Columbia, which do not report coccidioidal infections to the CDC. Surprisingly, Texas is one of the states that do not report cases of coccidioidomycosis despite its well-known endemicity (8–11). Although these are contributing factors, the CDC attributes the greatest degree of underreporting per capita to occur in Arizona and California, where coccidioidomycosis is reportable. In both states, cases are defined by positive laboratory results, most frequently serologic tests, and do not require clinical corroboration. If tests are positive, laboratories efficiently communicate new cases to public health, and lack of laboratory reporting is not a significant source of underreporting. Serologic tests for coccidioidomycosis are occasionally falsely negative, and when that occurs, a case might be missed. However, the most important reason for underreporting in these two states is the failure of clinicians to obtain the needed serologic tests on patients with coccidioidomycosis. The authors noted one study of Arizona internal medicine clinics which found that less than 13% of pneumonia patients were tested for coccidioidomycosis (12). In a study from Southern California, the testing percentage was 6% (13), and in Arizona emergency departments, it was 2.8% (14), complementing the experience in the authors’ own institution (15). This striking lack of serologic testing for coccidioidomycosis in pneumonia patients has persisted despite the abundant documentation of the problem and underscores the difficulty of changing clinician behavior (16–18).

The Valley Fever Center for Excellence at the University of Arizona College of Medicine-Tucson is now affiliated with a large health care system (Banner Health), affording the opportunity to initiate a systems approach within an organization to improve testing. Over the past several years, we have improved the rate of testing pneumonia patients in Banner urgent care clinics from less than 2% to greater than 40% (19), and we are now applying the same approach to the 14 Banner emergency departments within the endemic regions of Arizona. By making these improvements known to our colleagues, we hope to encourage them to do the same thing.

**Fig 1:**
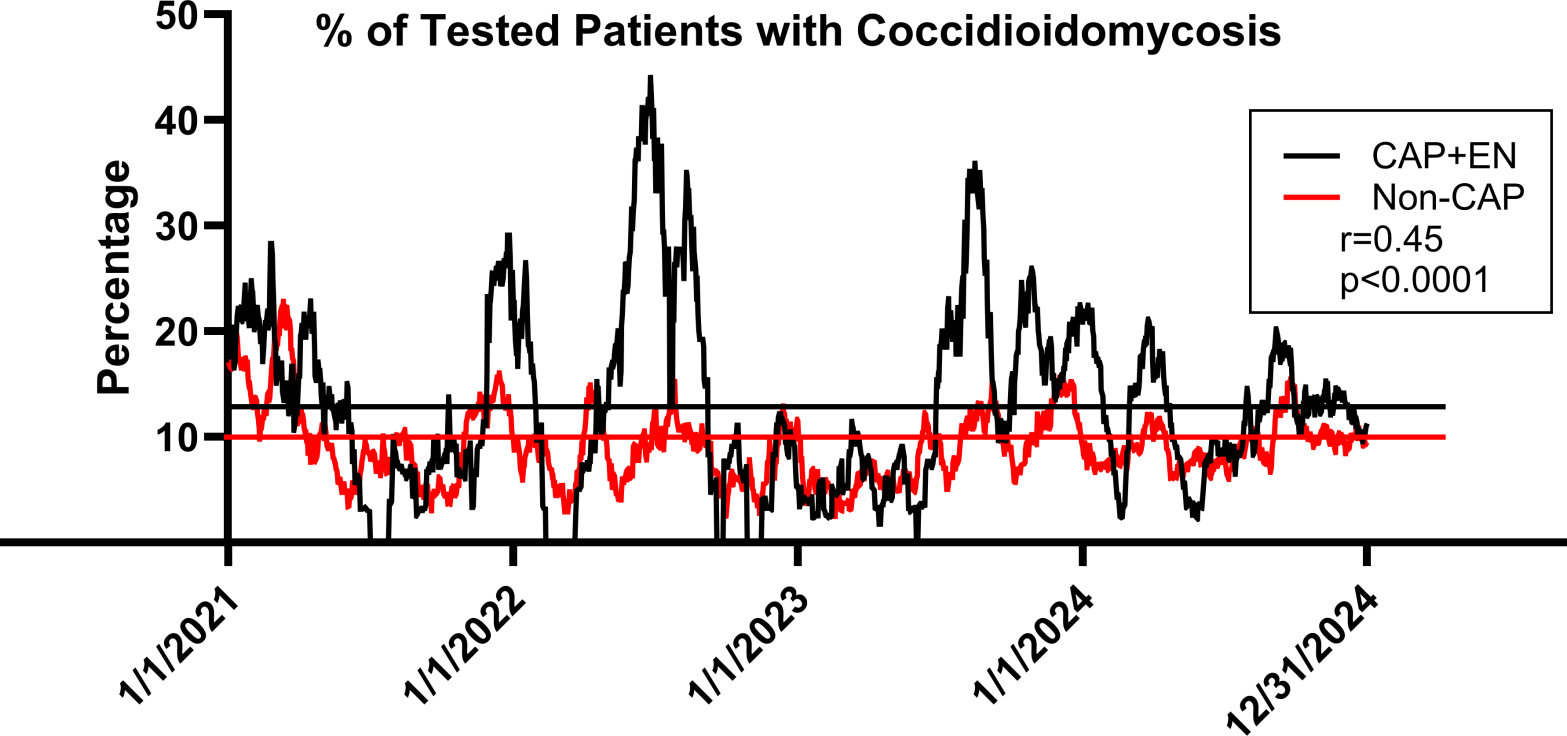
The graph represents 30 day moving averages of the percentage of urgent care patients whose coccidioidal serologic tests were positive. The black curve is for patients whose clinic visit included ICD-10 codes for either community-acquired pneumonia (J18.*) and/or erythema nodosum (L52). The red curve is for patient visits coded for neither. The horizontal lines indicate the average percent positivity for each curve using the same color.

An unexpected finding from the analyses of testing practices for coccidioidomycosis within Banner Health is that more cases of coccidioidomycosis were being diagnosed in patients who did not have the International Classification of Diseases, 10th Revision (ICD-10) code for community-acquired pneumonia (CAP) (J18.*) (19, 20). A few of these patients had erythema nodosum (ICD-10 code of L52), which in Arizona is highly associated with coccidioidomycosis. However, erythema nodosum is not a common skin condition and only accounted for a small proportion of those coccidioidomycosis patients without a pneumonia diagnosis. We know that newly infected patients have a variety of symptoms, including diffuse arthralgias, fatigue, and other rashes besides erythema nodosum (21, 22). Also, when respiratory symptoms are present, they may not lead to coding as pneumonia. None of these symptoms are very specific, and all are frequently seen in ambulatory practices everywhere. For patients with CAP and/or erythema nodosum in Maricopa County urgent care clinics over the past 4 years, the percentages of those tested for coccidioidomycosis that were positive ranged from 0% to 44% (19), depending upon current and prior weather patterns (23), with the overall average being 13% (Fig. 1). During the same period, the number of urgent care patients without either CAP or erythema nodosum numbered over 960,000, of which only 4 per 1,000 patients were tested for coccidioidomycosis. For those tested, the positivity rate ranged from 2.5% to 23.0%, a spread not nearly as great as for CAP patients, but the average rate that the tests were positive was 10%, surprisingly close to that for patients for whom the Infectious Diseases Society of America’s practice guidelines recommend testing (24). Also, there is a modest but significant temporal correlation between the two curves (*r* = 0.45, *P* < 0.0001). These similarities between the two curves may indicate that the clinicians who chose to test patients without pneumonia for coccidioidomycosis seem to have a sense of who to test. The task at hand is to figure out how to define it for everyone else.

In summary, for persons living in or visiting endemic regions for coccidioidomycosis, the possibility of contracting this illness is very much a fact of life. Physicians need to remember this when their patients have the typical signs and symptoms, even though those presentations overlap with other common diseases. Also, for patients with puzzling manifestations, such as the patient in this case report, adding coccidioidomycosis to the differential is appropriate. Since specific testing is needed to establish a diagnosis of coccidioidomycosis, remembering that this disease exists is the first step.
